# Assessment of the Impact of Temperature on Biofilm Composition with a Laboratory Heat Exchanger Module

**DOI:** 10.3390/microorganisms9061185

**Published:** 2021-05-31

**Authors:** Ingrid Pinel, Renata Biškauskaitė, Ema Pal’ová, Hans Vrouwenvelder, Mark van Loosdrecht

**Affiliations:** 1 Department of Biotechnology, Faculty of Applied Sciences, Delft University of Technology, Van der Maasweg 9, 2629 HZ Delft, The Netherlands; renata.biskauskaite@gmail.com (R.B.); ems.palova@gmail.com (E.P.); J.S.Vrouwenvelder@tudelft.nl (H.V.); M.C.M.vanLoosdrecht@tudelft.nl (M.v.L.); 2 Water Desalination and Reuse Center (WDRC), Division of Biological and Environmental Science and Engineering (BESE), King Abdullah University of Science and Technology (KAUST), Thuwal 23955-6900, Saudi Arabia

**Keywords:** heat exchange, biofouling, cooling tower, next generation sequencing, bacterial communities, PCOA, water quality, extracellular polymeric substances, industry, surfaces

## Abstract

Temperature change over the length of heat exchangers might be an important factor affecting biofouling. This research aimed at assessing the impact of temperature on biofilm accumulation and composition with respect to bacterial community and extracellular polymeric substances. Two identical laboratory-scale plate heat exchanger modules were developed and tested. Tap water supplemented with nutrients was fed to the two modules to enhance biofilm formation. One “reference” module was kept at 20.0 ± 1.4 °C and one “heated” module was operated with a counter-flow hot water stream resulting in a bulk water gradient from 20 to 27 °C. Biofilms were grown during 40 days, sampled, and characterized using 16S rRNA gene amplicon sequencing, EPS extraction, FTIR, protein and polysaccharide quantifications. The experiments were performed in consecutive triplicate. Monitoring of heat transfer resistance in the heated module displayed a replicable biofilm growth profile. The module was shown suitable to study the impact of temperature on biofouling formation. Biofilm analyses revealed: (i) comparable amounts of biofilms and EPS yield in the reference and heated modules, (ii) a significantly different protein to polysaccharide ratio in the EPS of the reference (5.4 ± 1.0%) and heated modules (7.8 ± 2.1%), caused by a relatively lower extracellular sugar production at elevated temperatures, and (iii) a strong shift in bacterial community composition with increasing temperature. The outcomes of the study, therefore, suggest that heat induces a change in biofilm bacterial community members and EPS composition, which should be taken into consideration when investigating heat exchanger biofouling and cleaning strategies. Research potential and optimization of the heat exchanger modules are discussed.

## 1. Introduction

Heat exchangers constitute a crucial part of process equipment such as cooling towers, where it is used to transfer the heat from a primary fluid to cold water [1]. Biofilm formation in heat exchangers can have strong negative impacts on process efficiency and is, therefore, considered a problematic challenge faced in numerous industrial sites. The dependence of industrial processes to heat exchangers makes the interruption of operation very difficult and, in many cases, requires continuous operation for several years before cleaning or replacement of parts can be performed [2]. Despite the use of biocides and dispersants, biofilms still develop over long periods of time and considerably reduce heat transfer efficiency. Additionally, they can serve as a protective habitat for pathogenic bacteria or bacteria involved in microbially induced corrosion, causing irreversible damage to the equipment [3,4]. These detrimental consequences strongly affect the capital and operational costs of the heat exchange installations [5,6].

Early investigations on biofilm resistance to heat transfer revealed a thermal conductivity of biofilm of 0.6 W/m·K [7], comparable to water, which is not surprising since water forms the major mass fraction of a biofilm [8]. Many studies have used this key parameter for monitoring of biofilm growth and assessment of control methods in heat exchanger experiments [9,10,11]. Tian et al. used this approach to evaluate the impact of SiO_2_ particles inclusion on biofilm structures and heat transfer resistance [12]. It was also used by Chang et al. to investigate the efficiency of thermal shock on biofilm growth inhibition [9].

A potential important aspect for biofilm growth is the presence of a temperature gradient over the length of the heat exchanger affecting the bulk water temperature. Bacterial communities composing the biofilm and producing extracellular polymeric substances (EPS) necessary for the biofilm integrity are subjected to this temperature change and may adapt. There is, however, limited literature available on its effect on biofilm composition since most research studies were performed under isothermal conditions [13,14,15]. Among the few studies considering the impact of thermal gradient, Yang et al. have shown that co-current or counter-current configurations of heat exchangers cause different fouling behaviors [16]. Undoubtedly, change in temperature exerts a strong influence on the extent and physical characteristics of biofilms. Due to these observed changes, it is reasonable to assume that variations in the bacterial communities and extracellular compounds also occur along heat exchangers. Characterizing the variations in biofilm composition is of importance to identify or predict which parts of heat exchangers are more detrimentally affected by the biofilm, and to optimize cleaning methods accordingly.

Laboratory scale heat exchanger set-ups have been designed in the past [17,18,19] to predict the impact of operational variables on biofouling rates for full-scale applications. These laboratory elements are, however, not suitable for biofilm collection and composition analyses. A compact plate heat exchanger module, inspired from a well-established membrane fouling simulator [20], was, therefore, designed and built to enable the growth of biofilms of significant thickness and characterization of its components. In this chapter, we describe (i) the development of this new single-plate heat exchanger module for biofilm laboratory investigations, (ii) its implementation to study the effect of temperature on the bacterial community and EPS composition of biofilms, and (iii) the potential applications and limitations of the module in biofilm characterization studies.

## 2. Materials and Methods

### 2.1. Laboratory Heat Exchanger Module

**Figure 1:**
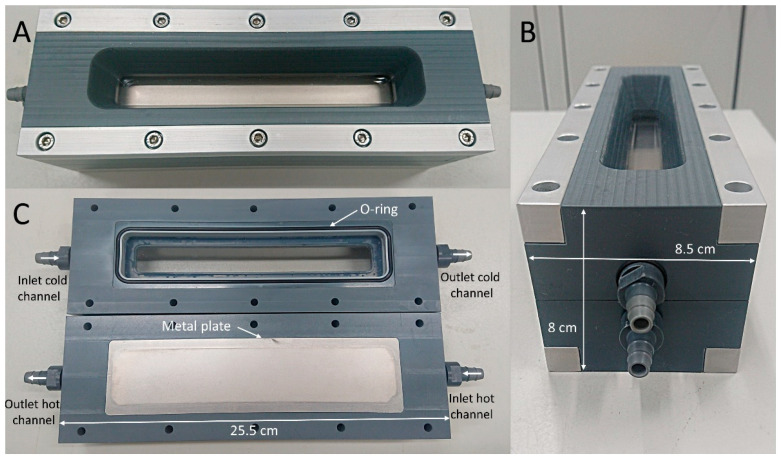
Heat exchanger module observed from the top side (**A**), from the left side (**B**), and view of the inside of the module with a rubber O-ring and metal plate (**C**).

The laboratory heat exchanger modules were designed as single plate heat exchangers. This design was selected due to its convenience for laboratory experiments, allowing easy access to cold and hot channels, and visual monitoring. The flat plate surface facilitates collection of biofilms. Modules were made of two identical PVC elements, fitting each other, and separated by a single metal plate (Figure 1). PVC connectors built in each side of the channels allowed connections to tubing. Diffusers were included in the geometry of the channels to provide a homogeneously distributed flow. O-rings were placed on each side of the metal plate to tightly maintain the plate and separate the water from each channel. Corrosion-resistant metal, Hastelloy C-22, was used for the metal plate. L × W × H dimensions were as follows: 189 × 34 × 8 mm^3^ for the channels and 189 × 34 × 1 mm^3^ for the plate. Two identical modules were produced by STT Products BV and operated simultaneously during our experiments. The technical drawing of the modules is provided in the Supplementary Materials (Figure S1).

### 2.2. Experimental Design

**Figure 2:**
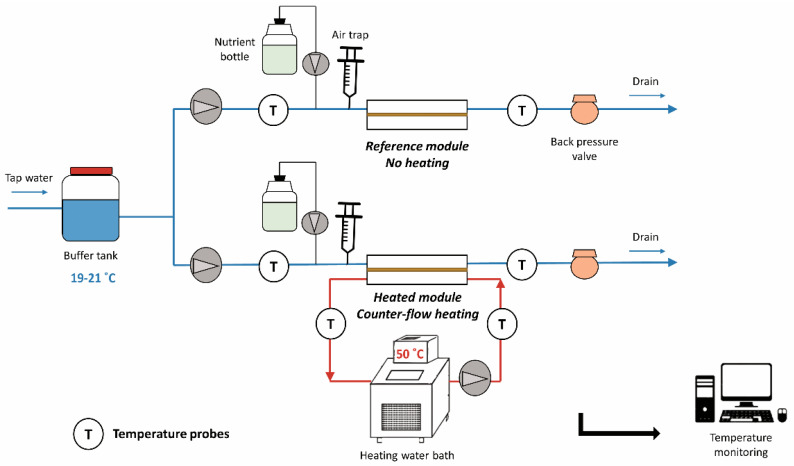
Schematic diagram of the heat exchanger set-up in operation.

In all experiments, one module was operated in single-pass flow without heating, denoted as “reference module”, and a second module was operated in counter-flow with continuous heating, denoted as “heated module”. The cold channel of each module was fed with tap water at a temperature of 20.0 ± 1.4 °C and a flow rate of 0.18 L/min. A nutrient solution composed of sodium acetate trihydrate (CH_3_COONa·3H_2_O; for a ‘C’ source), sodium nitrate (NaNO_3_; for a ‘N’ source) and sodium phosphate monobasic monohydrate (NaH_2_PO_4_·H_2_O; for a ‘P’ source) was dosed to enhance biofilm growth. The concentrations in elements C, N and P were maintained at 500, 100 and 50 µg/L, respectively, in the feed water, corresponding to a mass ratio C:N:P of 100:20:10 [21]. The second channel of the heated module only was operated with recirculating water at 50 °C at a flow rate of 1.8 L/min, without nutrient dosage. The full laboratory set-up is illustrated in Figure 2. Biofilms were grown over a period of 40 days. The experiments were performed in triplicate, denoted as “Experiments 1, 2 and 3”.

### 2.3. Temperature Monitoring

Platinum resistance thermometers of high-precision (CTP5000 WIKA, Klingenberg am Main, Germany) were located inside the inlet and outlet tubing of the channels, with a 10-cm deep immersion. Temperature probes were calibrated and showed 0.04 °C accuracy. All probes were connected to a display unit (CTR2000, WIKA, Klingenberg am Main, Germany) and data were recorded online via a data logging system on a local computer.

Heat transferred (*Q*) to the cold channel in module 2 was calculated with Equation (1):

(1) $$\begin{array}{c} Q=m*Cp*dT \end{array}$$

*m*: mass flow rate of water (L/s)

*Cp*: heat capacity of water—4184 J/(kg·K)

With *dT = T_out_ − T_in_*

The overall heat transfer coefficient (*U*) was approximated with Equation (2):

(2) $$\begin{array}{c} U=\frac{Q}{A*LMTD} \end{array}$$

*A*: area of the plate

*LMTD*: counter-current logarithmic mean temperature difference

With $LMTD=\frac{\left(T_{h.in}-T_{c.out}\right)-\left(T_{h.out}-T_{c.in}\right)\;}{LN\left(\left(T_{h.in}-T_{c.out}\right)/\left(T_{h.out}-T_{c.in}\right)\right)}$

*Uref* was calculated as average *U* over a period of 1 day, without dosages of nutrients. *Uexp* was monitored during the experiments to approximated the resistance (*R*) to heat transfer caused by the biofilm:

(3) $$\begin{array}{c} R=\frac{1}{U_{exp}}-\frac{1}{U_{ref}} \end{array}$$

The approximated thickness (*t*) of the biofilm was then obtained with Equation (4):

(4) $$\begin{array}{c} t=R*\lambda \end{array}$$

with *λ*: thermal conductivity of biofilms (0.6 W/m·K)

### 2.4. Biofilm Collection

At the end of each experiment, both modules were carefully drained and disassembled to recover the biofilms from the surface of the plates. 1 × 2 cm^2^ areas were sampled from the inlet and the outlet and preserved at −20 °C before DNA extraction and 16S rRNA gene amplicon sequencing. The remaining biofilms were scraped from the plates, frozen at −80 °C and lyophilized before EPS extraction.

### 2.5. DNA Extraction and 16S rRNA Gene Amplicon Sequencing

The genomic DNA was extracted using the DNeasy UltraClean Microbial Kit (Qiagen, Hilden, Germany). Extraction was done following company’s standard protocol with addition of an alternative lysis step. This included a combination of 5 min of heat (65 °C) followed by 5 min of bead-beating on the filters for cell disruption on a Mini-Beadbeater-24 (Biospec, Bartlesville, OK, USA). Samples were sent to Novogene Ltd. (Hongkong, China) for amplicon sequencing of the V3-4 region of the 16S-rRNA gene (position 341–806) on an Illumina paired-end platform. The raw data were processed with the software Mothur v.1.40.5 (Ann Arbor, MI, USA) [22]. Raw sequences were quality filtered, aligned, checked for chimera and operational taxonomic units (OTUs) were generated based on 97% similarities after removal of singletons. The alignment and taxonomic classifications were performed using the SILVA database [23]. The representative OTU sequence of *Cupriavidus* was compared to the RefSeq NCBI database using the Basic Local Alignment Search Tool (BLAST) for species identification. Beta diversity measurement was assessed with principal coordinate analysis (PCoA) in Mothur v.1.40.5 using the thetaYC distance matrix. The spatial separations visualized in the PCoA are used to compare similarities and dissimilarities between samples.

### 2.6. EPS Extraction

The extraction of EPS was performed on the freeze-dried raw deposits. EPS was extracted at 80 °C in alkaline conditions, following a method previously described [24,25], and lyophilized. The freeze-dried EPS samples were kept in a dry environment before further analyses.

### 2.7. Fourier Transform Infrared Spectroscopy (FTIR)

FTIR spectra of the freeze-dried extracted EPS samples were carried out on a FTIR Spectrophotometer (PerkinElmer, Waltham, MA, USA) at room temperature, with a wavenumber range from 700 to 4000 cm^−1^. Resolution of 4 cm^−1^ and accumulation of 8 scans were applied on each sample. FTIR spectra were baseline-corrected and a min–max normalization was applied with respect to the amide I peaks (between 1700 and 1600 cm^−1^) with Matlab R2018b software (Mathworks, Natick, MA, USA).

### 2.8. Polysaccharide Quantification

Polysaccharides were quantified following a method developed by [26]. In short, the freeze-dried EPS extracts were diluted in a sodium hydroxide solution (0.01 M in ultrapure water) to a final concentration of 1000 mg/L. For the standards, a sugar mixture of 1000 mg/L was prepared with equal amounts of fucose, rhamnose, galactose, glucose, xylose, mannose, and ribose. Sugars were selected due to their previous detection in bacterial EPS [27]. The sugar mixture was diluted to concentrations of 0, 10, 25, 50, 75, 100, and 200 mg/L in order to establish the calibration curve. Then, 200 µL of each sample or standards were pipetted in a glass reaction tube, followed by 200 µL of 5% *w*/*v* phenol solution and 1000 µL of 95% sulfuric acid. Tubes were vortexed, left at room temperature for 20 min and vortexed again before analyses. The absorbance of the samples and standards were measured at 482 nm with a spectrophotometer (DR3900 Hach, Loveland, CO, USA).

### 2.9. Protein Quantification

The freeze-dried EPS extracts were diluted in a sodium hydroxide solution (0.01 M in ultrapure water) to a final concentration of 250 mg/L. Total protein concentrations were determined with the BCA assay using the Protein Quantification Kit (Interchim, Montluçon, France). The standards were prepared by dilution of a bovine serum albumin (BSA) solution to concentrations of 0, 5, 10, 50, 100, 200, 350, and 500 mg/L. Reagents were added to the standards and samples according to manufacturer’s instruction in a 96-well plate. The plate was shaken for 30 s and incubated in the dark at room temperature for 2 h before analysis. Absorbance was measured at 562 nm by a plate reader (Infinite M200 PRO Tecan, Männedorf, Switzerland).

## 3. Results

### 3.1. Operating Parameters

The reference module was operated with the cold channel only, without heating. Inlet and outlet measurements indicated negligible variation of the bulk water temperature over the channel length (Table 1). The cold channel of the heated module was operated with the same flow rate, feed source and nutrient dosage as the reference module throughout the experiments.

Water heated at 50 °C was recirculating in counter-flow through a second channel (Figure 1), along which a decrease of 1 °C was measured. The heated flow caused a heat transfer through the metal plate, increasing the bulk water temperature of the cold channel from 20.1 ± 1.3 °C to 27.3 ± 1.3 °C between the inlet and outlet. When no biofilm was present in the heated module, the calculated overall heat transfer coefficient reached 660 ± 40 W/m^2^ °C, which was used as the reference value for the later calculation of the biofilm heat resistance. Biofilm growth caused a reduction in the temperature difference between inlet and outlet by 1.6 ± 0.2 °C in the cold channel of the heated module. Collection of temperature data along the heated system allowed the monitoring of the biofilm thickness forming on the surface of the metal plate.

### 3.2. Monitoring of Biofilm Growth in the Heated Module

**Figure 3:**
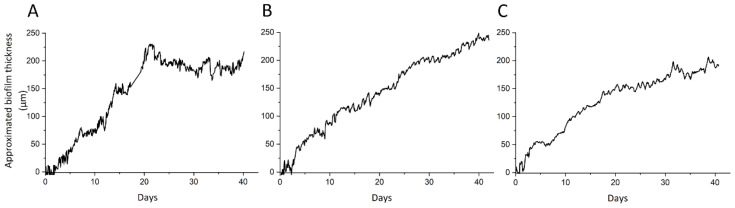
Approximated thickness of the biofilms monitored in the heated module over the duration of Experiment 1 (**A**), Experiment 2 (**B**), and Experiment 3 (**C**).

Approximated biofilm thicknesses were calculated with Equation (4) from online temperature measurements of inlets and outlets of each heated module. Biofilm growth curves of the heated modules are shown in Figure 3. Experiment 1 revealed disturbances due to intermittent air bubbles entering the system causing fluctuations in temperature. This anomaly was fixed in Experiment 2 and Experiment 3. Despite slight differences in profile, all experiments show a similar growth curve with a final thickness between 200 and 250 µm after 40 days of operation. Reproducibility of the study was achieved in terms of biofilm development in the heated heat exchanger module.

### 3.3. Biofilm Characterization

#### 3.3.1. Bacterial Community Structure along the Heat Exchanger

Accumulated biofilms were collected from the inlet and outlet of each module to evaluate the changes in bacterial communities along the heated and reference systems, and assess the effect of a temperature gradient.

**Figure 4:**
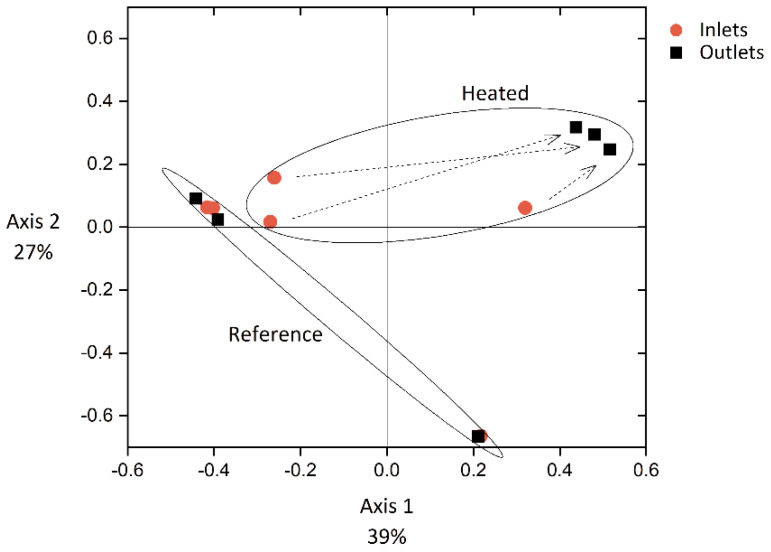
Principal coordinate analysis (PCoA) of biofilm bacterial community structures from the heated and reference modules in the three experiments. The bigger the distance between data points, the stronger the dissimilarity in community structures, based on the presence and abundance of operational taxonomic units. Dashed arrows connect inlet and outlet of each module.

Dissimilarities between the structure of bacterial communities are illustrated in Figure 4. Although a deviation is visible for a set of data points at coordinates (0.22, −0.64), corresponding to Experiment 3, inlet and outlet of the reference module showed high similarity in each experiment. Main identified groups in Experiments 1 and 2 references were *Variovorax* and *Piscinibacter* genera and *Microscillaceae* family. In Experiment 3, the genus *Aquabacterium* represented a large fraction of the total relative abundance (Figure S2). On the contrary, inlet and outlet of the heated module experienced a strong change in bacterial community structure in each experiment, represented in Figure 4 by dashed arrows. Although *Aquabacterium*, *Curvibacter*, *Microscillaceae,* and *Variovorax* groups were commonly identified in the inlets, *Cupriavidus* was dominating the outlets of all three heated modules. Therefore, changes observed along the metal plate in absence of heating were negligible compared to the changes that occurred under heated condition. The temperature gradient along the metal plate thus resulted in a change in biofilm composition in terms of bacterial community members and abundances in the heated module.

Interestingly, outlet biofilm samples of the heated module from the three experiments converge to a similar structure. Taxonomic identification indicated a significant increase in *Cupriavidus respiraculi* abundance, reaching 52%, 79%, and 58% in the heated outlet biofilms of Experiments 1, 2, and 3, respectively (Figure S2). Results suggest the loss in diversity of main community members at elevated temperatures, with the selection of *Cupriavidus respiraculi*.

#### 3.3.2. EPS Amounts

The total biofilm from each metal plate was collected to obtain sufficient EPS for further characterization. This allowed the comparison of the biofilm composition of the reference module to the biofilm composition of the heated module but not the evaluation of changes along the plates. The relative low amount of EPS per plate did not allow for sampling different areas of the plate separately. The average freeze-dried deposit collected in the reference module reached 10.8 ± 3.0 mg with an extracted EPS yield of 52 ± 2%. These values were not significantly different (*p*-values > 0.05) to the heated module, with 11.2 ± 2.6 mg and 57 ± 8% of EPS yield. In conclusion, similar amounts of accumulated biofilm and EPS were found in all systems.

#### 3.3.3. EPS Characterization

**Figure 5:**
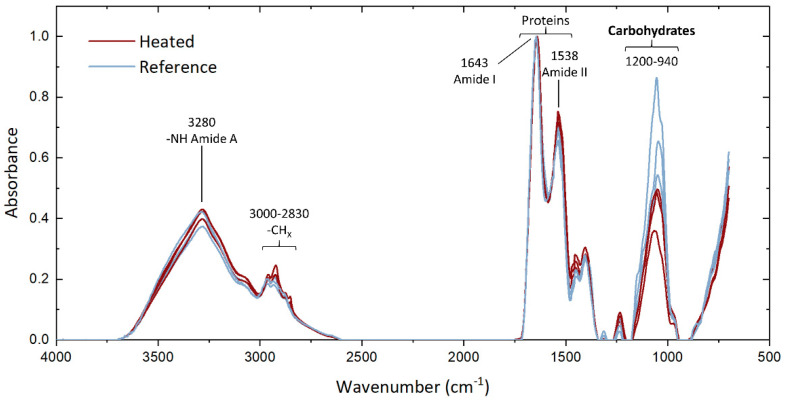
FTIR of the extracted EPS of the biofilm samples collected on the heated and reference modules. The band associated to carbohydrates at 940–1200 cm^−1^ shows a strong divergence between the heated and reference conditions [29].

The FTIR spectra of the extracted EPS samples display the protein band at 1500–1700 cm^−1^ with the peaks corresponding to C=O and C-N groups of amide I and N-H, C-N, and C-C groups of amide II [28]. The characteristic carbohydrate band is visible at 940–1200 cm^−1^, corresponding to stretching vibrations of C-O in C-OH and C-C group [29,30]. The comparison of spectra indicates high similarity in molecular vibrations with a large overlapping fraction of the curves of the extracted EPS from the heated and reference modules. Only the carbohydrate peak does not overlap (Figure 5), with a striking divergence between both conditions. A lower absorbance intensity is observed in the EPS grown under heated conditions, suggesting that a relatively lower sugar content of the EPS was produced at elevated temperature.

**Figure 6:**
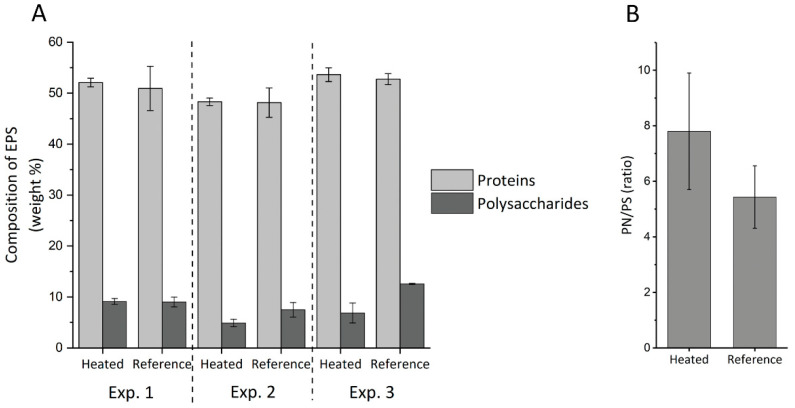
Composition of EPS in weight percentages of polysaccharides and proteins in the heated and reference modules (**A**) and protein-to-polysaccharide (PN/PS) ratio under both conditions, averaged over the triplicate experiments (**B**). The error bars indicate the standard deviations.

The quantitative analyses of protein and polysaccharide contents in the extracted EPS samples led to the results shown in Figure 6A. The percentage of proteins remained quite stable with variations between 48% and 54% of the total EPS, while polysaccharides constituted between 5% and 13% of the total EPS. As observed with the FTIR analyses, the reference EPS seemed to contain a higher amount of polysaccharide than the EPS from the heated module, especially in Experiment 2 (*p*-value < 0.05) and 3 (*p*-value < 0.05). The ratio of proteins to polysaccharides was therefore calculated for each module and the values from both tested conditions were compared. Despite a partial overlap in standard deviations, the statistical analysis indicates a significant difference (*p*-value < 0.05) between the conditions, with a higher PN/PS ratio in the EPS extracted from the heated biofilm, 7.8 ± 2.1% compared to 5.4 ± 1.0% in the EPS extracted from the reference biofilm (Figure 6B).

In summary, despite similarity in amounts of accumulated EPS in the reference and heated modules, EPS characterization reveals a small but significant change of protein to polysaccharide ratio caused by a relatively lower extracellular sugar production at elevated temperatures.

## 4. Discussion

### 4.1. Impact of Temperature on Biofilm Composition

The triplicate experiments performed with the newly designed modules led to reproducible results regarding biofilm growth profile (Figure 3) and EPS characterization (Figure 5 and Figure 6). Only the bacterial community structures showed a deviation in Experiment 3 compared to Experiments 1 and 2 (Figure 4), likely caused by the effect of external factors affecting the tap water planktonic community. Variation in external temperatures and overall water use in the building could have affected the bacterial community structure [31,32,33] since the experiments were performed sequentially. Despite this deviation, conclusions could be drawn from the results. They are further discussed below.

Experimental analyses of the biofilms grown in the reference and heated modules revealed (i) variations in biofilm community structures caused by the temperature increase along the plate heat exchanger, (ii) comparable amounts of accumulated EPS, and (iii) a relatively lower production of extracellular polysaccharides at elevated temperature by the biofilm members.

Abundant members of our study include bacterial groups frequently found in drinking water (Figure S2), such as the *Piscinibacter* genus [34], the *Aquabacterium* genus populating biofilm with optimal growth at 20 °C [35] or the *Variovorax* genus [36]. Their high abundance was particularly marked in the reference biofilms, without heating. Selection of the species *Cupriavidus respiraculi* occurred in the biofilm grown in the heated module (Figure S2). *Cupriavidus respiraculi* is a gram-negative, obligate aerobe bacterium which has been observed to grow at 28, 32, and 37 °C [37,38]. The species was isolated from patients with cystic fibrosis suggesting that they are well adapted for growth at physiological temperatures. Dominance of *Cupriavidus* over the drinking water biofilm groups with increasing temperature is an indication that the temperature change faced in heat exchangers leads to biofilm bacterial community compositions diverging from the feed water source communities. It is also important to note that the processes of detachment and dispersion or reattachment of biofilm fragments [39], observed in mature biofilms, did not seem to have affected the community structure of the heated biofilm in the 40-day studies.

These results reveal that the temperature gradient in heat exchangers causes the selection of different biofilm bacterial groups along the system, and can, therefore, cause damages of varying nature and magnitude along the process, e.g., regarding the extent of biofouling or microbial corrosion [40]. The effect of heat gradient should, therefore, be taken into account and samples should be collected along the system when characterizing biofilms from heat exchangers.

Analyses showed that the amounts of biofilms and accumulated EPS were not significantly different between the reference and the heated biofilms. It is surprising, since more severe biofouling is usually observed at higher temperatures [41,42]. Felz et al. has shown that EPS yield can be strongly affected by the method of EPS extraction [24]. However, the comparable protein content from both extracted EPS (Figure 6) does not suggest disparities in cell lysis by the alkaline extraction. Biofilm growth and EPS production in the heated biofilm might be limited by the carbon supply or by the wide temperature range along the plate and through the thickness of the biofilm. For a better assessment, the exact temperatures at the surface of the plate and through the biofilm should be monitored in future studies.

The quantitative measurements of proteins and sugars revealed a lower sugar content of extracted EPS from the heated biofilms (Figure 6), which is in line with the lower band intensity in the region 940–1200 cm^−1^ observed in the FTIR analyses (Figure 5). The change in temperature therefore induced variations in the produced extracellular compounds. These changes in EPS composition along heated surfaces can affect the efficiency of biofilm removal chemicals such as enzymatic treatments. Studies at larger scale and after long periods of heat exchanger operations are needed for comparison with our laboratory results. Sampling and EPS analyses from a fouled full-scale heat exchanger surface with wide temperature gradient would be of high interest to evaluate the extend of such variations and the implications from an industrial point of view.

### 4.2. Advantages and Limitations of the Heat Exchanger Laboratory Module

The purpose of the developed lab-scale heat exchanger module is to reproducibly investigate the impact of a continuous temperature gradient on biofilm formation, in terms of active biomass and EPS amount and composition. It was, therefore, designed according to characteristics required for carrying out the research study. Its laboratory-suited size makes it easy to assemble and disassemble and allows a flexible use of the device in various locations. The height of the channel was chosen so that it can sustain the growth of a thick biofilm without causing a build-up in pressure or significant variation in fluid velocity which could eventually affect the biofilm development, e.g., through an increase in shear rate [43,44]. Stable conditions could, thus, be maintained during the operating period. The material used for the metal plate (hastelloy C-22) was selected for its anti-corrosion properties and to allow an optimal heat transfer from the hot to cold channel of the module—hastelloy has a thermal conductivity of 11.1 W/m·K—while the external case was made of PVC to limit heat loss. In addition, the plate can easily be replaced to test different materials, which can broaden the spectrum of potential studies to (microbiologically influenced) corrosion investigations.

There are, however, some limitations to the heat exchanger set-up. A further developed version of the module should include online measurements of plate surface temperatures and investigation of the temperature profile over the height of the biofilm. The temperature at the surface of the plate is generally higher than the bulk temperature and is affected by the boundary layer, which could be approximated by modeling of the system. Oxygen profile probes would also provide information on the oxygen diffusion through the layer. Design could be enhanced by using material able to handle higher temperatures than PVC can resist, while maintaining an acceptable heat loss. For temperatures higher than 50 °C, it is recommended to change material to thermoplastics with high heat deflection temperature such as polyethersulfones (PES). Lastly, some loss of biofilm was observed during the drainage of the cold channels leading to a small loss in biofilm materials. The handling of this step could be improved in order to reach a full recovery of the grown biofilm.

### 4.3. Potential Applications in Future Research

When operated over a sufficiently long period of time, the system can be used to perform studies on biofilm properties and compositions that would not be possible with biofilms of a few µm-thickness. The testing of a broader range of temperatures and the use of additional chemical characterization methods of EPS would build on the results compiled in this study. In addition to the preliminary results described in this research, we also propose some study lines of interest, which could be tested with the laboratory plate heat exchanger module.

Regarding the characterization of the microbiome, variations can be assessed along the temperature gradient, as introduced in this study. Investigation of the bacterial community changes could also be performed across the thickness of the biofilm [45], by collecting layers of the biofilm formed between the metal plate and the bulk water. The differences in main bacterial group, their physiology and interactions would provide a deeper understanding of how the biofilm is able to cope with the significant changes in conditions, not only linked to the diffusion of nutrient and oxygen [46] but also the effect of thermal dissipation.

The applied monitoring provides an approximated thickness, assuming a flat and homogeneous structure of the biofilm over the plate surface. Flow profile, temperature, and nutrient load can, however, affect the growth and the distribution of the biofilm [47]. In addition, it has been seen in pure-culture biofilm that temperature can cause a regulation of EPS production inducing morphological changes of the biofilm [15]. To evaluate this assumption accurately, the temperature monitoring should be combined with measurement of the thickness. For more in-depth investigation of the physical structure, in-situ imaging of the biofilm would provide valuable information on the biofilm development and morphology (e.g., roughness) when subjected to a thermal gradient, in a similar way as it was performed for different types of flow [48].

Due to the adjustability of the module, the use of metal plate prone to corrosion such as copper or carbon steel can be explored. Microbiologically influenced corrosion causes serious damages in full-scale plants [49], and is very challenging to control due to the combination of processes involved in the corrosion mechanisms [50]. The module can for example be used for the testing of metals or innovative coatings, and assessment of their ability to withstand microbial corrosion under different temperatures.

In short, the heat exchanger can be used for a multitude of research purposes. The fact that thick biofilms can be grown in the module and collected in substantial amounts allow the use of multiples biofilm analyses for in-depth characterization of its composition.

## 5. Conclusions

A laboratory-scale plate heat exchanger module for the assessment of the impact of temperature gradient on biofilm composition was built, tested and applied to a preliminary biofilm investigation.

The online monitoring and analyses showed that:

- the developed plate heat exchanger was able to monitor online biofilm growth by measuring its resistance to heat transfer;
- the laboratory module is suitable for a large range of applications related to the effect of a thermal field, such as bacterial community identification through the height of the biofilm, composition and morphology of EPS at various temperatures, or microbial corrosion investigations.

The uniqueness of the module lies in its suitability to sustain and monitor extensive biofilm formation with negligible effect on flow properties, and to allow collection of sufficient biofilm material to perform destructive analyses of composition. Some suggestions based on the exploratory study were proposed for further improvement of the module.

Exploratory study on the effect of temperature gradient revealed:

- comparable amounts of biofilm and accumulated EPS formed in the non-heated and heated systems over the 40-day experiments;
- differences in proteins-to-polysaccharides ratio in extracellular polymeric substances caused by the thermal field, with a lower production of polysaccharides at elevated temperature;
- differences in biofilm bacterial groups resulting from the temperature change at the surface of the plate.

## Figures and Tables

**Table 1:** Operational parameters monitored during the operation of the modules, with the interval (±) representing the range of variations. Indicated values combine the data of all experiments.

Module	Temperature Hot Channel (°C)		Temperature Cold Channel (°C)		Flow Rate Hot Channel (L/h)	Flow Rate Cold Channel (L/h)	Heat Loss (%)
	Inlet	Outlet	Inlet	Outlet			
Reference	r.t. *	r.t. *	20.0 ± 1.4	20.1 ± 1.2	0	11.4	approx. 0
Heated	50.4 ± 0.2	49.3 ± 0.2	20.1 ± 1.3	27.3 ± 1.3	108	11.4	27 ± 3

* r.t.: room temperature.

## Data Availability

The sequencing data presented in this study are openly available at doi:10.4121/14667879.

## Supplementary Materials

The following are available online at https://www.mdpi.com/article/10.3390/microorganisms9061185/s1, Figure S1: Technical drawings of the heat exchanger module showing the top/bottom view (top), the longitudinal cross-section (middle) and the transverse cross-section (bottom). Figure S2: Relative abundances of bacterial genera in biofilm samples collected from the reference and heated modules in Experiment 1 (A), Experiment 2 (B), and Experiment 3 (C). Phyla and classes of Proteobacteria are indicated at the right of the legend. Experiments were performed in triplicate.
