# Supplementary material for: Assessment of the Impact of Temperature on Biofilm Composition with a Laboratory Heat Exchanger Module
Source: Microorganisms. 2021 May 31;9(6):1185. doi: 10.3390/microorganisms9061185 (PMC8229324; doi:10.3390/microorganisms9061185)
Supplement: Supplementary file 1 [file microorganisms-09-01185-s001.zip › microorganisms-1229662-supplementary.pdf]

Supplementary Information:

Assessment of the Impact of Temperature on Biofilm Composition with a Laboratory Heat Exchanger Module

Figures S1 and S2

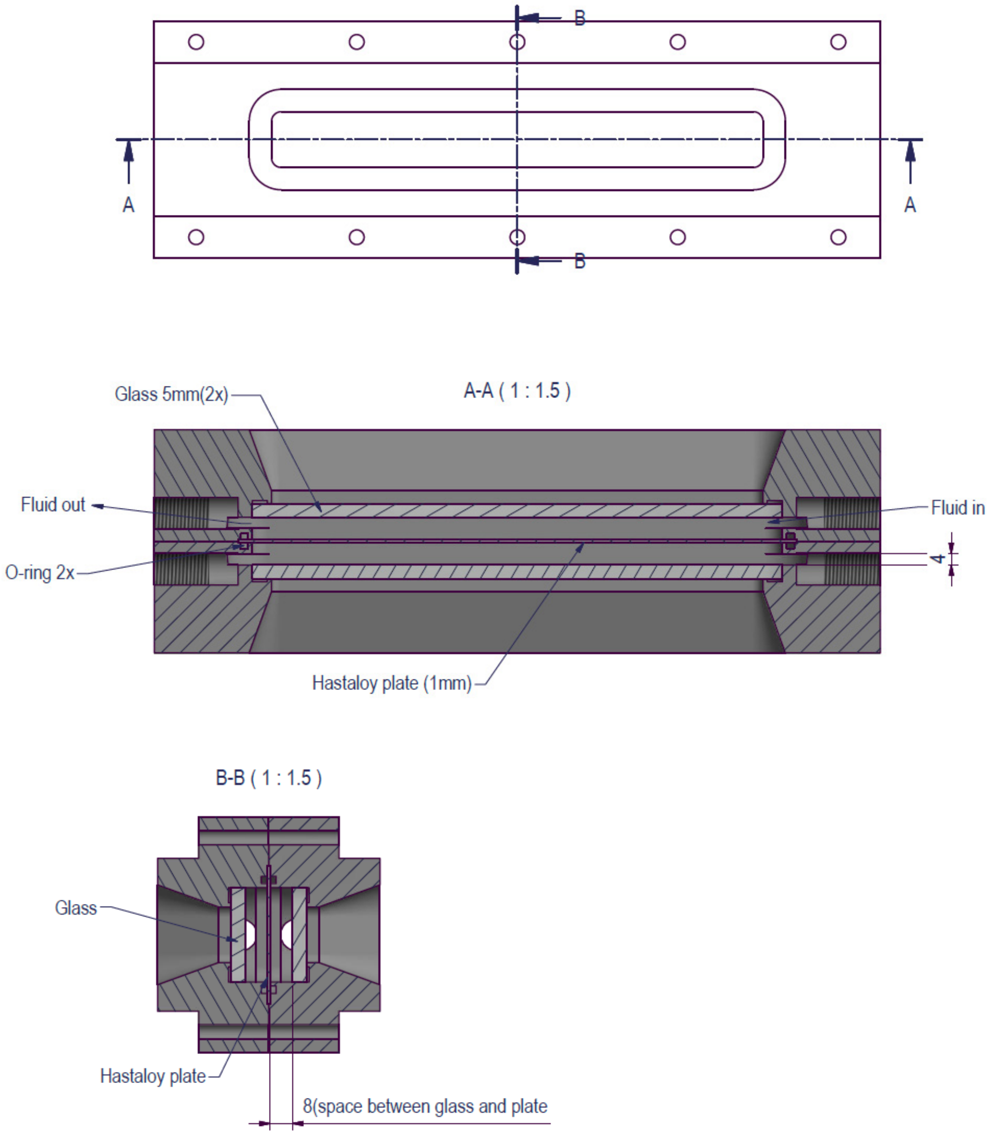

Figure S1. Technical drawings of the heat exchanger module showing the top/bottom view (top), the longitudinal cross-section (middle) and the transverse cross-section (bottom).

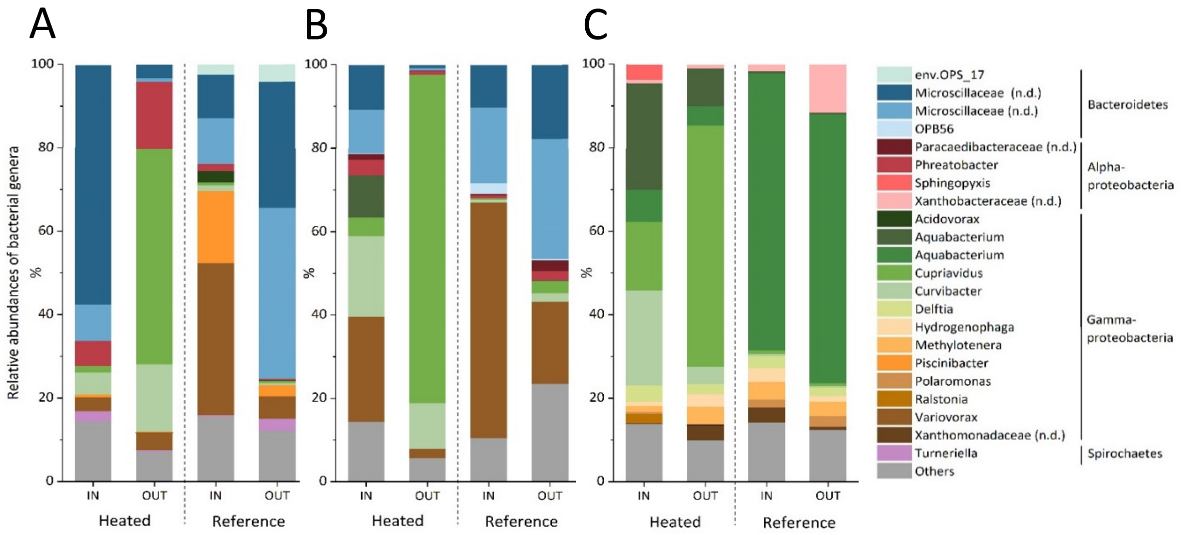

Figure S2. Relative abundances of bacterial genera in biofilm samples collected from the reference and heated modules in exp. 1 (**A**), exp. 2 (**B**), and exp. 3 (**C**). Phyla and classes of Proteobacteria are indicated at the right of the legend. Experiments were performed in triplicate.
